# Supplementary material for: Multi-scale modeling of intensive macroalgae cultivation and marine nitrogen sequestration
Source: Commun Biol. 2021 Jul 7;4:848. doi: 10.1038/s42003-021-02371-z (PMC8263761; doi:10.1038/s42003-021-02371-z)
Supplement: Supplementary file 2 — Supplementary Information [file 42003_2021_2371_MOESM2_ESM.pdf]

Supplementary Information for

Multi-Scale Modeling of Intensive Macroalgae Cultivation and Marine Nitrogen Sequestration

Meiron Zollmann[1]*, Boris Rubinsky[2], Alexander Liberzon[3] and Alexander Golberg[1].

[1]Porter School of Environmental and Earth Sciences, Tel Aviv University, Tel Aviv, Israel
[2]Department of Mechanical Engineering, University of California at Berkeley, Berkeley, CA, US
[3]School of Mechanical Engineering, Tel Aviv University, Tel Aviv, Israel.

Supplementary Information Text

Supplementary Methods

Model dimensions. The multi-scale model is a 3D model, as described in **Supplementary Figure 1**. x axis is the direction of the flow, along which cultivation reactors are chained, y axis is the horizontal direction perpendicular to the flow, along which multiple rows of reactors can be placed, and z axis points downwards from the water surface. Only one layer of reactors can be placed along the z axis, as sun exposure is necessary.

Step-by-step multi-scale model formulation.
Ulva metabolism model. Our multi-scale model was developed based on a mathematical model of metabolism of the *Ulva* sp. macroalgae, modified from previous works[1]. The model assumes that the dynamics of the limiting nutrient, in this case nitrogen (N), under the constraining effects of major environmental conditions (light intensity (I), temperature (T) and salinity (S)) predicates the dynamics of biomass growth and chemical composition. The model follows the Droop Equation concept, relating growth to internal nutrient concentrations ("cell quota") rather than to the external, environmental, concentrations[2,3]. A schematic description of this metabolism model is presented in **Supplementary Figure 2.** The model is based on three governing equations, describing the mass balance of three state variables: biomass density (m, g Dry Weight (DW) l^{-1}, eq S1), biomass internal nitrogen (N_{int}, $\%\ \mathrm{g\ N\ kg\ DW^{-1}}$, eq S2) and external nitrogen in water (N_{ext}, $\mu\mathrm{mol\ N}\ l^{-1}$, eq S3), under varying temperatures, light intensities and salinities. The set of governing ordinary differential equations (ODEs) is summarized in **Supplementary Table 1.** Further details and reasoning are presented below**.**
Growth Rate. The specific growth rate of the algae is a function of I, T, S and nutrients[1]. In the marine environment, the limiting nutrient is usually N[4] and our model focuses on N limited environments. However, similar models can be developed also for other elements such as phosphorus (P) and ferrous (Fe) that may limit growth in some marine environments. Following Liebig's law of the minimum, we formulate the growth rate as a function of the limiting factor, which is the scarcest resource in relation to the macroalgae's requirement. This formulation is based on a simplifying assumption that there are no interactions between these factors and thus no co-limitations. However, temperature and salinity effects were excluded from the minimum law, as they constitute background conditions for metabolic or uptake efficiency and are not considered as growth resources. Therefore, the approximated function for biomass growth rate appears in eq S1.1. We adjusted daily specific growth and losses rates to hourly rates, assuming for simplicity that growth and biomass losses occur only during light hours (see details below). This assumption ignores night growth that occurs due to metabolites produced during light-time photosynthesis[5], and thus distorts growth distribution along the day. However, it has no effect on total daily growth and therefore does not impair the model accuracy in resolutions of days to weeks.

$$\mu = \mu_{max} f, f = f_{Temp} f_S \min\{f_{N_{int}}, f_{P_{int}}, f_I\} \tag{S1.1}$$

Where μ_{max} (h^{-1}) is the maximum specific growth rate and f is the combined growth function, made of f_{Temp}, f_S, $f_{N_{int}}$, $f_{P_{int}}$ and f_I which are the temperature, salinity, internal N, internal P and light intensity growth functions.

Biomass Losses. The specific rate of biomass losses due to respiration, exudation and mortality is a function of temperature, and is calculated relatively to a known losses rate in a reference temperature. We used the losses function from Martins and Marques[6]. This does not include losses by grazing, sporulation events and fragmentation by storms, which vary significantly between different environments and are highly affected by extreme events.

$$\lambda = \lambda_{20}\,\theta^{T-20} \tag{S1.2}$$

Where λ_{20} (h^{-1}) is the specific rate of biomass losses and θ is an empiric factor of biomass losses.

Temperature Effects. Temperature affects all metabolic processes and thus also growth rate. We adapted the temperature function from Martins and Marques[6] with minor adjustments, setting the exponent n as a free parameter, as opposed to the original $n = 2$.

$$f_{Temp} = \exp\left(-2.3\left(\frac{T - T_{opt}}{T_x - T_{opt}}\right)^n\right) \tag{S1.3}$$

Where $T_x = T_{min}$ for $T \le T_{opt}$ and $T_x = T_{max}$ for $T > T_{opt}$. T_{min}, T_{opt} and T_{max} (℃) are the minimal, optimal and maximal temperatures for *Ulva* growth.

Salinity Effects. Similarly, salinity has a general effect on nutrient uptake and growth of macroalgae. However, the effect of salinity on *Ulva* is relatively minor, as its flexible cell membrane allows it to adjust to a wide range of salinities[7]. Here, we used the salinity growth function from Martins and Marques[6] and adjusted it to fit to high salinities by changing the exponent of the original function (from $b = 2$ to $b = 4.4$) to achieve a minimal error compared to measured data from Choi et al.[7]

$$f_S = 1 - \left(\frac{S - S_{opt}}{S_x - S_{opt}}\right)^b \; for\; S \ge 5 \; or \; \frac{S - S_{min}}{S_{opt} - S_{min}} \; for\; S < 5 \tag{S1.4}$$

Where $S_x = S_{min}$ and $b = 2.5$ for $S < S_{opt}$, and $S_x = S_{max}$ and $b = 4.4$ for $S \ge S_{opt}$. S_{min}, S_{opt} and S_{max} (PSU) are the minimal, optimal and maximal salinities for *Ulva* growth.

Light Effects. Light intensity effect on growth is described by eq S1.5[8]. A more precise description of light effects will relate also to the photosynthesis process, that occurs only during light time but can result in production of new biomass also at night. For simplicity, we formulate a direct effect of light on growth, and normalized daily growth rate to per light-hour growth rate, thus minimizing the effect on model results (See growth rate section).

$$f(I) = \frac{I}{K_I + I} \tag{S1.5}$$

Where I is the incident light intensity and K_I (μmol photons m^{-2} s^{-1}) is the light half saturation constant.

Nutrients Effects. The model assumes that at each time point growth is limited by a single factor, which is a single nutrient or the light intensity. To simplify the model, we assume that both ambient and internal N:P ratio are smaller then 12, and therefore N is the limiting nutrient and $f_{P_{int}}$=1 [1]. Internal N and light intensity effects on growth are described in eq S1.6[9]. Internal N effects can be divided into three ranges: $N_{\text{int}} = N_{\text{int min}}$ where there is no growth as reserves are empty and all N_{int} is structural N, $N_{\text{int min}} < N_{\text{int}} < N_{\text{int crit}}$ where growth rate increases as N_{int} increases, and $N_{\text{int crit}} < N_{\text{int}}$ where further filling of the reserves does not increase growth rate[10,11]. It should be noted that we preferred this formula over the common Michaelis-Menten equation[1] as the range of N_{int} in the biomass is limited, and the mathematical requirement of neglecting the half growth rate constant in high N_{int} values ($K_N \ll N_{int,max}$) cannot be fulfilled.

$$f_{N_{int}} = \frac{\frac{N_{\text{int}} - N_{\text{int min}}}{N_{\text{int}}}}{\frac{N_{\text{int crit}} - N_{\text{int min}}}{N_{\text{int}\,crit}}} \text{ for } N_{int} < N_{\text{int crit}}, \text{ or } f_{N_{int}} = 1 \text{ for } N_{int} > N_{\text{int crit}} \quad \text{(S1.6)}$$

Where $N_{\text{int min}}$ and $N_{\text{int max}}$ (% g N g DW^{-1}) are the minimum and maximum internal nitrogen concentrations in *Ulva*, respectively, and N_{crit} (% g N g DW^{-1}) is the threshold N_{int} level below which the growth rate slows down.

Nutrient Reserves. Following the assumption of N limitation, internal N is the only modelled reserve. An underlying assumption is that the organic carbon reserve, depending on carbon uptake and photosynthesis rates, is not limiting within the modelled conditions (temperature ranges, seaweed density and water exchange and aeration rates). N_{int} is affected by uptake of external N ($\psi_{N_{ext}}$, eq 2.1) and by dilution by growth ($N_{\text{int}}\mu$m).

$$\psi_{N_{ext}} = \frac{N_{\text{int max}} - N_{\text{int}}}{N_{\text{int max}} - N_{\text{int min}}} \frac{V_{\max} N_{\text{ext}}}{K_S + N_{\text{ext}}} \quad \text{(S2.1)}$$

Where $V_{\max}$ (μmol N g DW^{-1} h^{-1}) is the maximum N uptake rate and K_S (μmol N l^{-1}) is the N half saturation uptake constant.

External Nutrients. N_{ext} represents the N concentration in the water in a cultivation reactor and is best described by the Convection–Diffusion equation[12] (eq S3.1).

$$\frac{\partial N_{ext}}{\partial t} = \nabla \cdot (D \nabla N_{ext}) - \nabla \cdot (\text{v} N_{ext}) - \psi_{N_{ext}} m \quad \text{(S3.1)}$$

Where D (m^2 s^{-1}) is the average diffusivity coefficient of dissolved inorganic nitrogen species and v (m s^{-1}) is the velocity field in which the dissolved nitrogen is moving.

This equation can be simplified into eq S3, following a few assumptions: 1. D is constant in space; 2. velocity flow is incompressible, and 3. net diffusivity is zero, as the reactor is well-mixed and there is no concentration gradient ($\nabla N_{ext} = 0$). Therefore, N_{ext} in the reactor is affected only by the N supply by airlift pump (normalized to reactor volume) and N uptake by the algae.

Model upscaling. We upscaled the model in a two-step manner, as described in **Figure 1**. On the first step, the metabolic model which applies to a single thallus scale (1 cm) is transitioned into a reactor scale (1 m) by adding an average light distribution term[8], allowing to adjust light intensity per mass to biomass density changes (eq S4). In this equation we multiplied I_0 by a 0.43 PAR constant, representing the ratio of the sunlight which is suitable for photosynthesis[13]. Each reactor is assumed to be well-mixed by bottom aeration and is connected to an airlift pump, that supplies the reactor with fresh sea water and nutrients, as described by Chemodanov et al. [14]. We assume water flow through reactor boundaries is negligible.

$$f(I) = \frac{I_{average}}{K_I + I_{average}}, I_{average} = \frac{I_0\, PAR}{K_0 Z + K_a SD}\left[1 - \exp\left(-(K_0 Z + K_a SD)\right)\right] \quad \text{(S4)}$$

Where $I_{average}$ (μmol photons m^{-2}s^{-1}) is the average photon irradiance in the reactor, I_0 (μmol photons m^{-2} s^{-1}) is the incident photon irradiance at water surface, SD (gDW m^{-2}) is the Stocking Density of biomass per unit of water surface in the reactor, K_0 (m^{-1}) is the water light extinction coefficient, Z (m) is the maximum water depth in the reactor, K_a (m^2 gDW^{-1}) is the *Ulva* light extinction coefficient and PAR is the Photosynthetically Active Radiation ratio of sunlight.

On the second step, the reactor scale model is transitioned into a farm scale model (1 km), simulating a row of reactors which are chained along the flow. Thus, each reactor constitutes a N sink, causing the spatial change of N concentrations in the environment. By assuming the width of this change is small in respect to the distance between the rows, a quasi-2D approximation can be made, making this model applicable also to multiple rows of reactors. This step requires the addition of a fourth governing equation, describing N dynamics in the environment, which is the connecting agent between the different reactors (eq S5). Each reactor is connected to the

environment by the inflow of the airlift pump and the equal outflow of water surplus (eq S6), and the change in the environmental N concentrations depends on stream flow (Q_s) and environmental N concentrations (N_{env}), dilution ratio (*d*), pumping flow (Q_P) and uptake ($\psi_{N_{ext}}$) in reactors. All four ODEs were solved numerically with hourly time steps.

$$\frac{\partial N_{env}}{\partial t} = \frac{\left[-\mathbf{Q_s}(N_{env}(1-d)-N_{env})-\mathbf{Q_p}\left(N_{env}-N_{ext_x}\right)\right]}{\boldsymbol{V_{cage}}} \quad \text{(S5)}$$

Initial and boundary conditions: $N_{env} = N_{env_{(x,t=0)}} = N_{env_0}$

$$\frac{\partial N_{ext}}{\partial t} = \frac{\mathbf{Q_p}\left(N_{env_x}-N_{ext_x}\right)}{\boldsymbol{V_{cage}}} - \psi_{N_{ext}}\, m, \text{ IC: } N_{ext_{(x,t=0)}} = N_{env_0} \quad \text{(S6)}$$

Calculation of hourly growth and losses rates. Maximum specific daily growth and losses rates were adjusted to hourly rates using eq S7. The formula relates to μ_{max} but can be used in the same way for λ_{20}. The equation was solved using the Microsoft Excel Office 365 solver.

$$m_0(1 + \mu_{day,max}) = \sum_{i=0}^{\text{light hours}} m_i(1 + \mu_{hour,max}) \quad \text{(S7)}$$

Where m_0 is the initial biomass density, m_i is the biomass density in hour i, $\mu_{day,max}$ is the maximum specific daily growth rate, light hours is the number of daily light hours and is set to 14 according to empiric data and $\mu_{hour,max}$ is the maximum specific hourly growth rate.

Model simulations. *Simulated environmental conditions*. **Supplementary Figure 5** presents water temperature and light intensity profiles used for model simulations. Light intensity data was extracted from the IMS data base from the Israel Meteorological Services (https://ims.data.gov.il/he/ims/6). Ex-situ light intensity data was obtained at a 3-hour resolution and interpolated to a 1-hour resolution. Water temperature data was measured in-situ in a 1-month resolution and interpolated to a 1-hour resolution[15]. **Supplementary Table 3** summarizes the main environmental data used for simulations and model boundary conditions. This data was collected and analyzed by Suari et al.[15] in a comprehensive study of the Alexander estuary. Salinity was set in a constant value of 30 PSU for two reasons. First, we found that in the examined range the model is not sensitive to the salinity level. Second, the study of Suari et al.[15] found complex estuary salinity dynamics, controlled by sandbar breaches rather than by seasonal phenomena, that do not allow describing salinity variations in an annual timeline manner.

Supplementary Results

Model calibration results.

In- vs Ex-situ light intensity data and model projections. In-situ light intensity and temperature data was obtained in a 15-minute resolution and averaged to a 1-hour resolution. Ex-situ light intensity data was obtained at a 3-hour resolution and interpolated to a 1-hour resolution. Temperature and light intensity profiles of returns 2, 4 and 5, followed by optimized models for each return based on both data sets, are presented in **Supplementary Figures 6-8.** Temperature and light intensity profile of returns 1, followed by an optimized model based on both ex-situ data is presented in **Supplementary Figure 9.** The in-situ based modelling uses eq S1.5 and the ex-situ based modelling uses eq S4.
In- vs Ex-situ light intensity data parametric distribution. $RMSRE_1$ was calculated for 320 parametric combinations of μ_{max}, K_a, K_I, n, T_{max}, T_{opt} and λ_{20} in a defined range (**Supplementary Table 4**). Plotting $RMSRE_1$ vs the examined parameters (**Fig. S11**), points out K_a as the only parameter with a clear trend, achieving minimal errors around $K_a = 0.15$.
Next, after setting K_a as 0.15, $RMSRE_2$ was calculated for 280 parametric combinations of μ_{max}, K_I, n, T_{max}, T_{opt} and λ_{20} in the same defined range (**Supplementary Table 4**). Plotting $RMSRE_2$ vs these 320 examined parametric combinations (**Supplementary Figure 11**) suggests setting λ_{20} and K_I on the low side of the examined range. Therefore, and after manual examination of specific parametric combinations, we set $K_I = 20$ and $\lambda_{20} = 0.0016$.

Model parameters. Details regarding model parameters, including original values, examined ranges and chosen values, are presented in **Supplementary Table 4**. **Supplementary Table 5** presents the detailed calibration results, comparing measured and modeled biomass production in returns 1,2,4 and 5, and average and specific relative errors.

Model simulations. *Seasonal trends in biomass production and nitrogen removal.* **Supplementary Figure 12** presents how shortening the cultivation cycles during spring and autumn can enable maintaining a constant chemical composition year-round.

Supplementary Figure 1. Dimensions of the multi-scale model

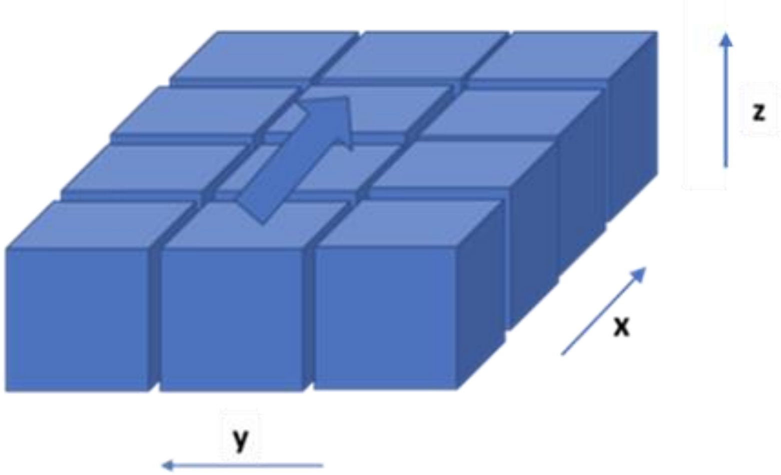

x axis is the direction of the flow, along which cultivation reactors are chained, y axis is the horizontal direction perpendicular to the flow, along which multiple rows of reactors can be placed, and z axis points downwards from the water surface. Only one layer of reactors can be placed along the z axis, as sun exposure is necessary.

Supplementary Figure 2. A schematic description of the metabolic model of *Ulva*.

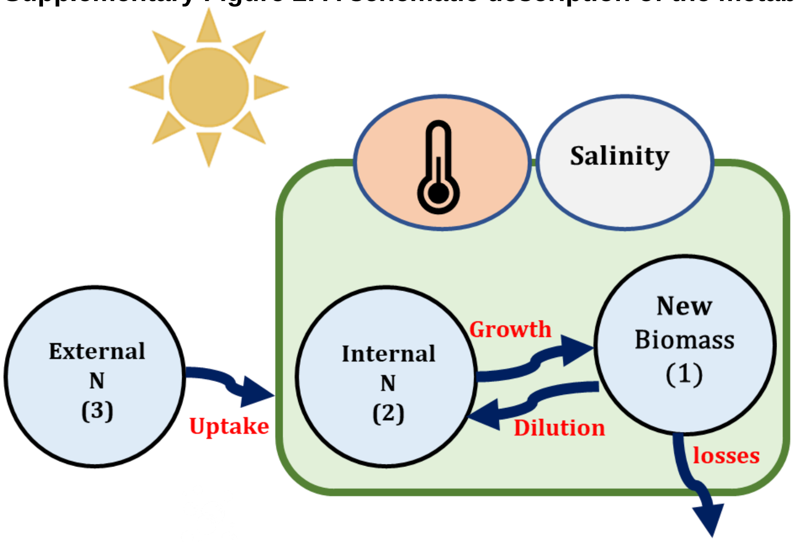

The rounded rectangle represents the algae. The surrounding area represents the environment to which the algae is exposed. Circles represent the model state variables and constraining environmental conditions are represented by ellipses and icons: light intensity with a direct effect on growth rate and salinity and temperature that affect all biological processes.

Supplementary Figure 3. Design of the cultivation reactor simulated in this study and map of cultivation site

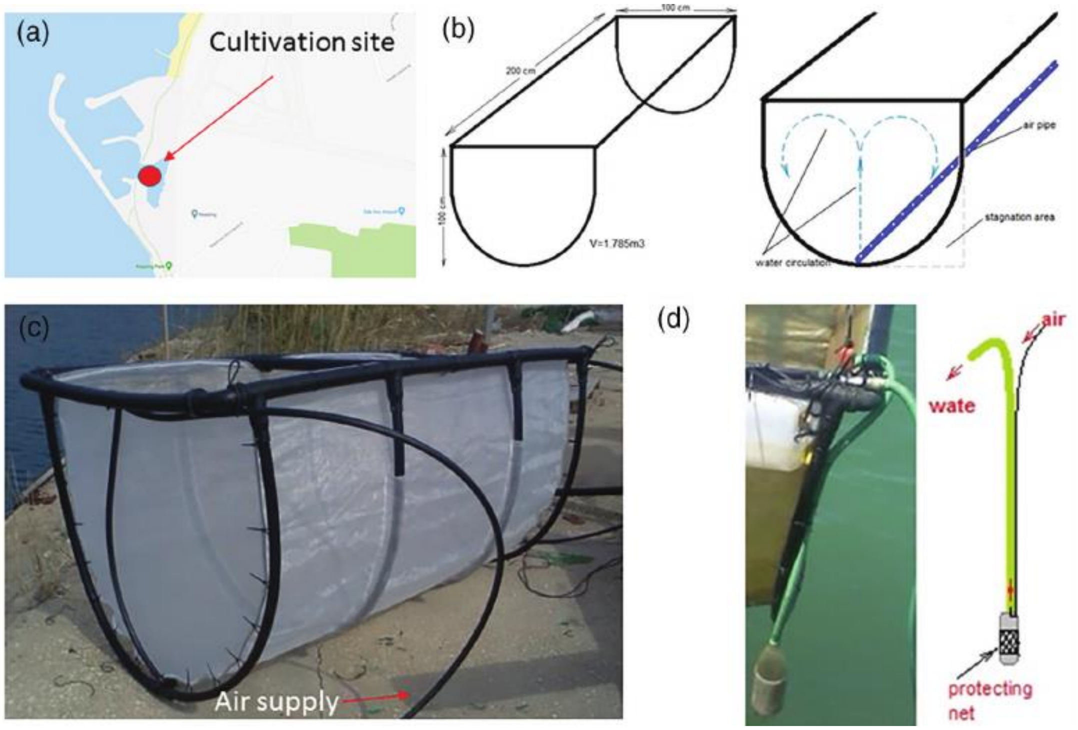

(a) Cultivation site; (b) schematic design of the reactor with intensification with tumbling, mixing and water exchange; (c) image of the reactor for intensified cultivation, and (d) external airlifts for water exchange enhancement. (Adapted from Chemodanov et al. [14] with permission).

Supplementary Figure 4. Cultivation reactor with biomass

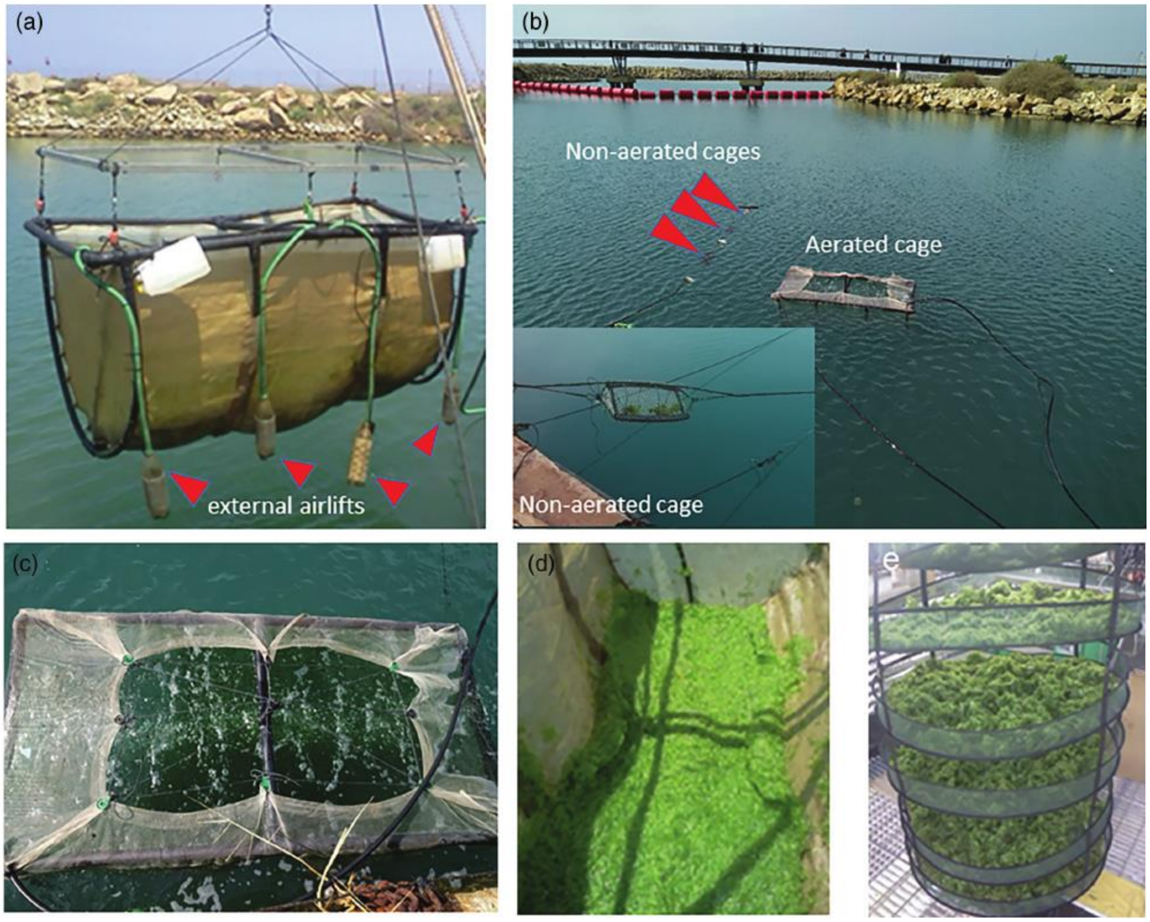

(a) Image of the cultivation reactor with external airlifts; (b) deployment of the reactor with algae to the cultivation site; (c) tumbling with air and mixing of *ulva* sp. biomass in the reactor; (d) harvested *Ulva* biomass after water removal with gravitation and (e) solar dried *Ulva* biomass (Adapted from Chemodanov et al. [14] with permission).

Supplementary Figure 5. Environmental conditions of the simulated cultivation environment

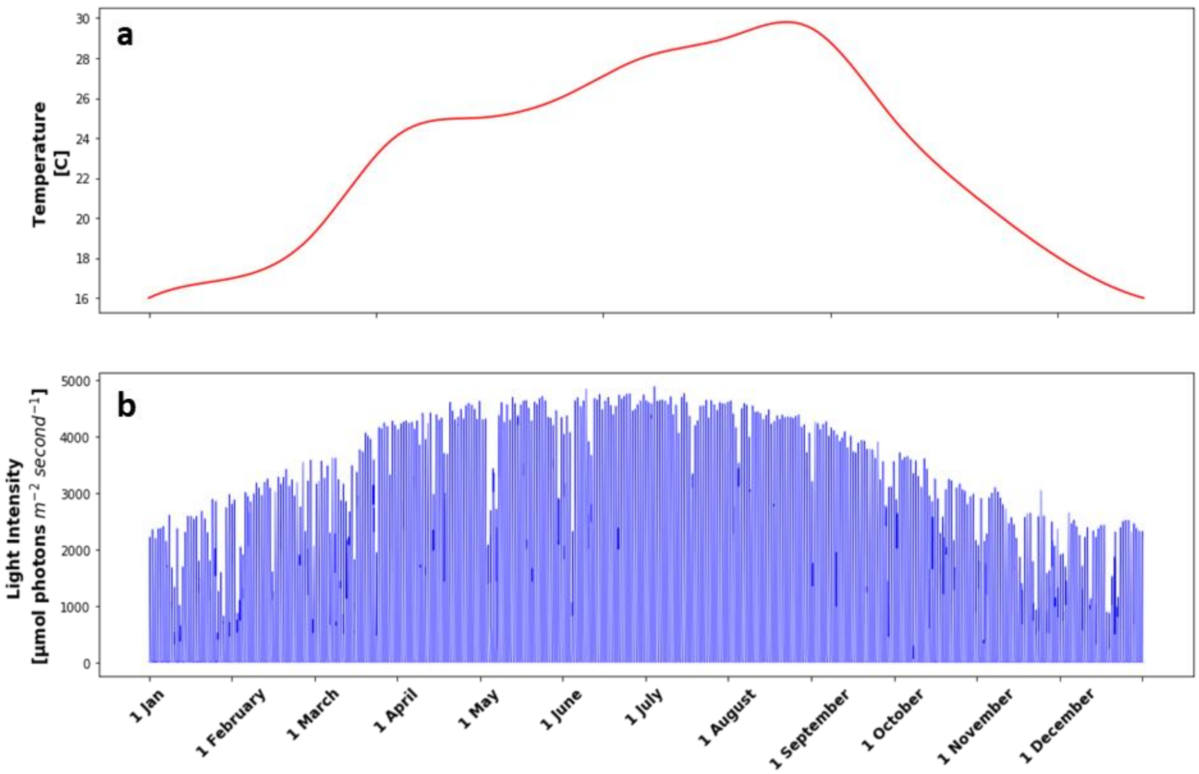

(a) Water temperature and (b) light intensity profiles of the simulated environment.

Supplementary Figure 6. Environmental conditions and calibration simulations of return 2.

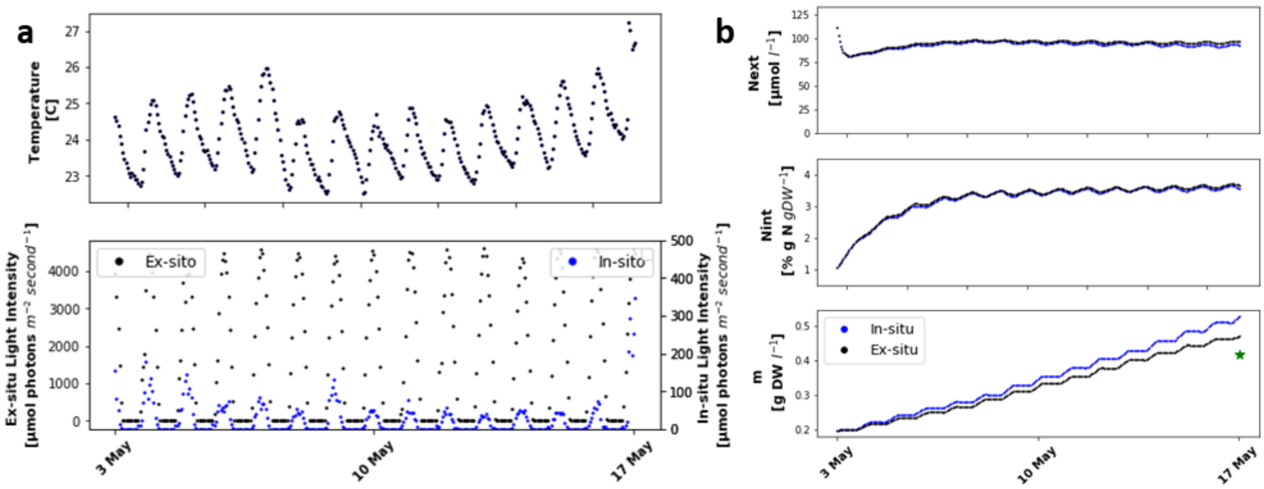

(a) Temperature and light intensity profile. (b) Model results based on in-situ (blue) and ex-situ (black) light intensity measurements. Green star marks measured final *m*.

Supplementary Figure 7. Environmental conditions and calibration simulations of return 4.

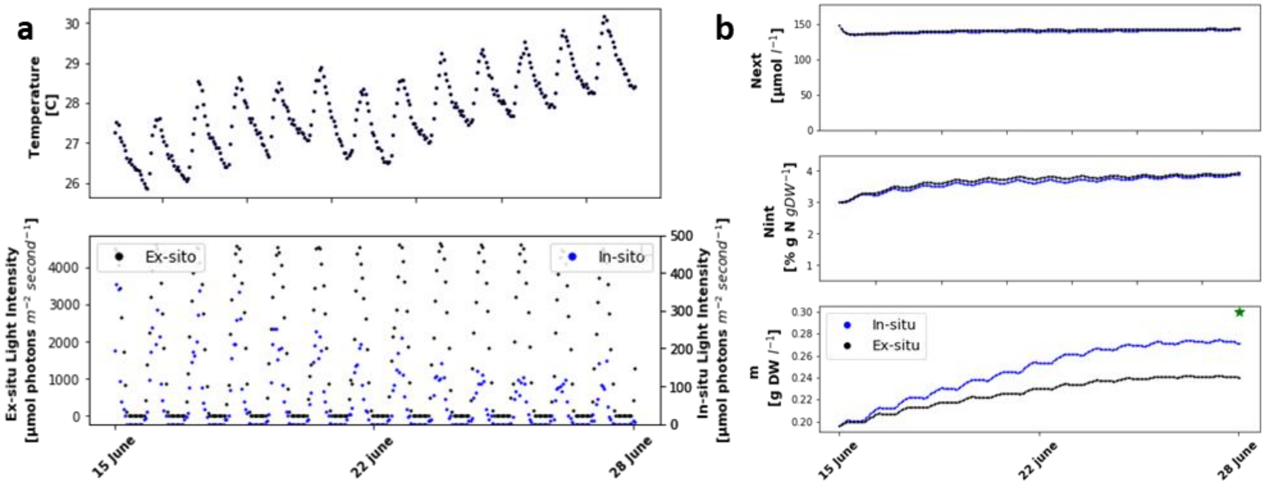

(a) Temperature and light intensity profile. (b) Model results based on in-situ (blue) and ex-situ (black) light intensity measurements. Green star marks measured final *m*.

Supplementary Figure 8. Environmental conditions and calibration simulations of return 5.

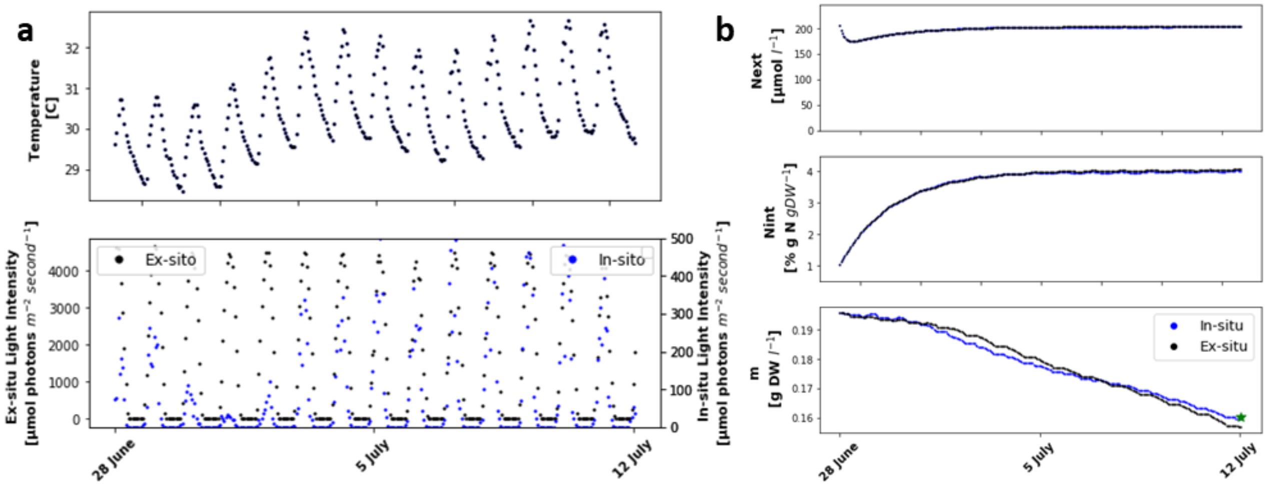

(a) Temperature and light intensity profile. (b) Model results based on in-situ (blue) and ex-situ (black) light intensity measurements. Green star marks measured final *m*.

Supplementary Figure 9. Environmental conditions and calibration simulations of return 1.

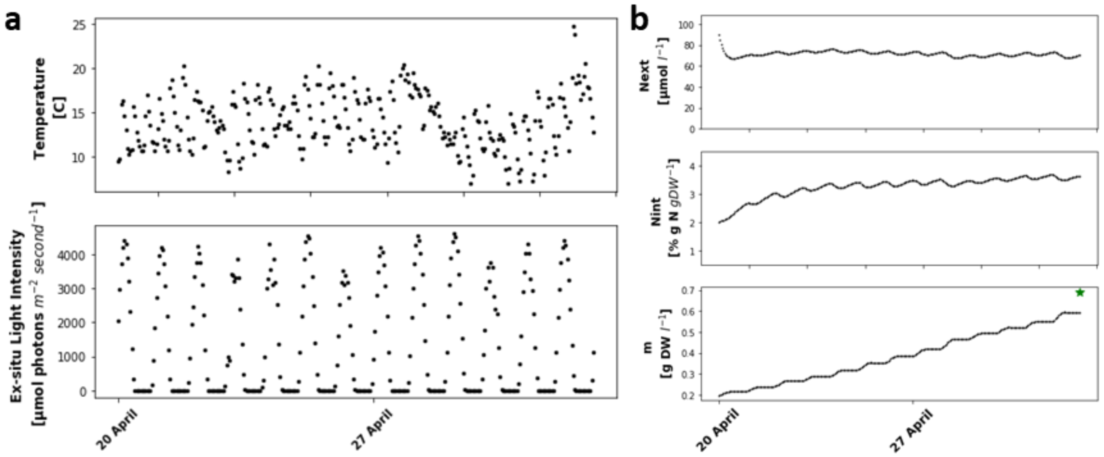

(a) Temperature and light intensity profile based on ex-situ measurements. (b) Model results based on ex-situ temperature and light intensity measurements. Green star marks measured final *m*.

Supplementary Figure 10. Distribution of root mean relative errors between modelled biomass growth based on in situ measured light intensity and experimental results

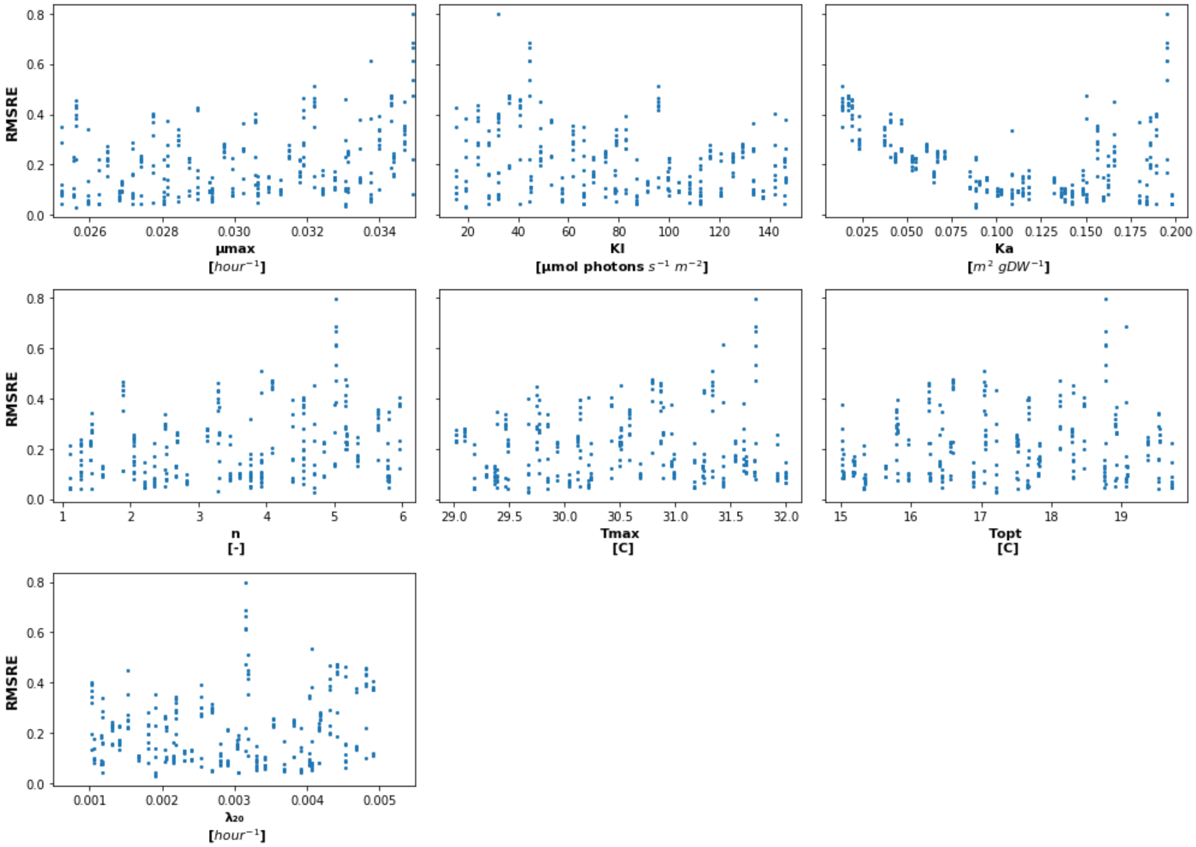

$RMSRE_1$ distribution for parametric combinations of μ, λ_{20}, K_a, K_I, n, T_{max} and T_{opt} in a defined range (See **Supplementary Table 2**).

Supplementary Figure 11. Distribution of root mean relative errors between modelled biomass growth based on light intensity data extracted from the Israel Meteorological Services databased and experimental results

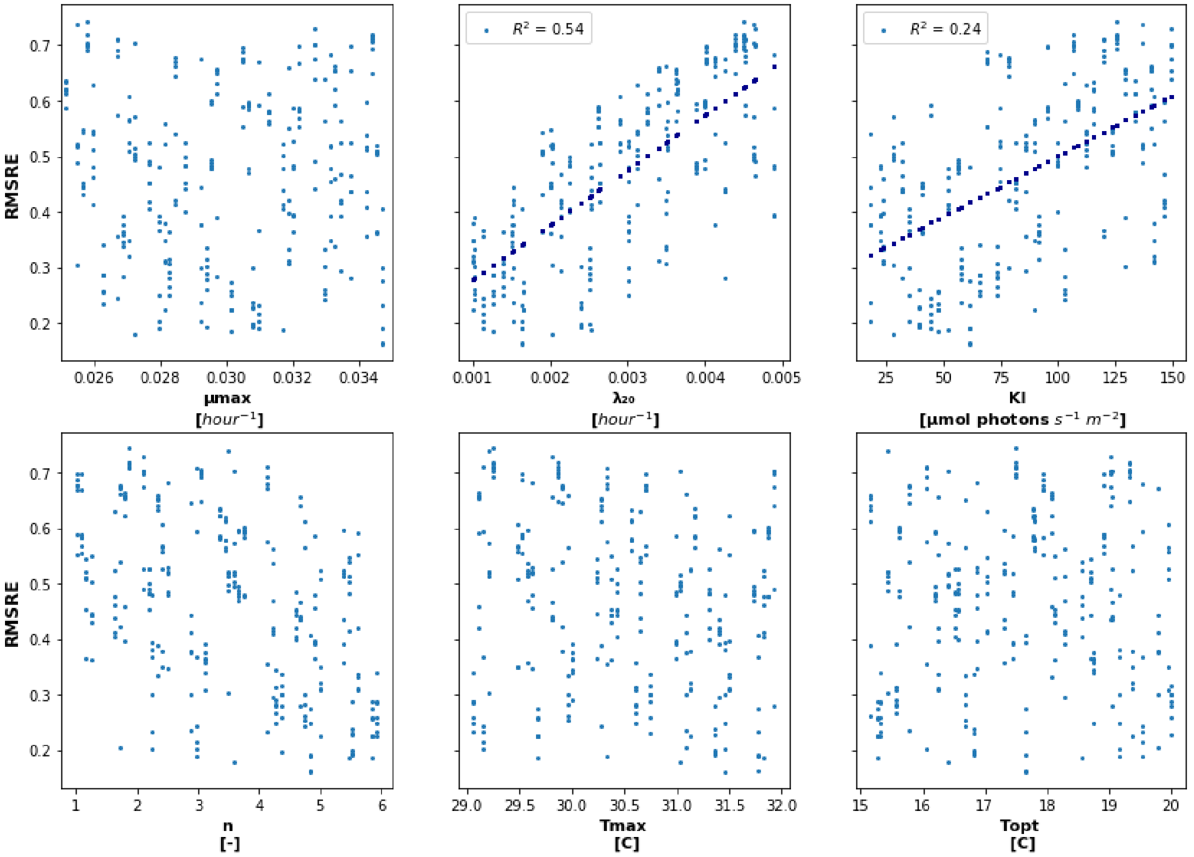

$RMSRE_2$ distribution for parametric combinations of μ, λ_{20} , K_I, n, T_{max} and T_{opt} in a defined range (See **Supplementary Table 2**).

Supplementary Figure 12. N_{env}, N_{int} and m dynamics along cultivation periods adjusted for uniform internal N contents in different seasons.

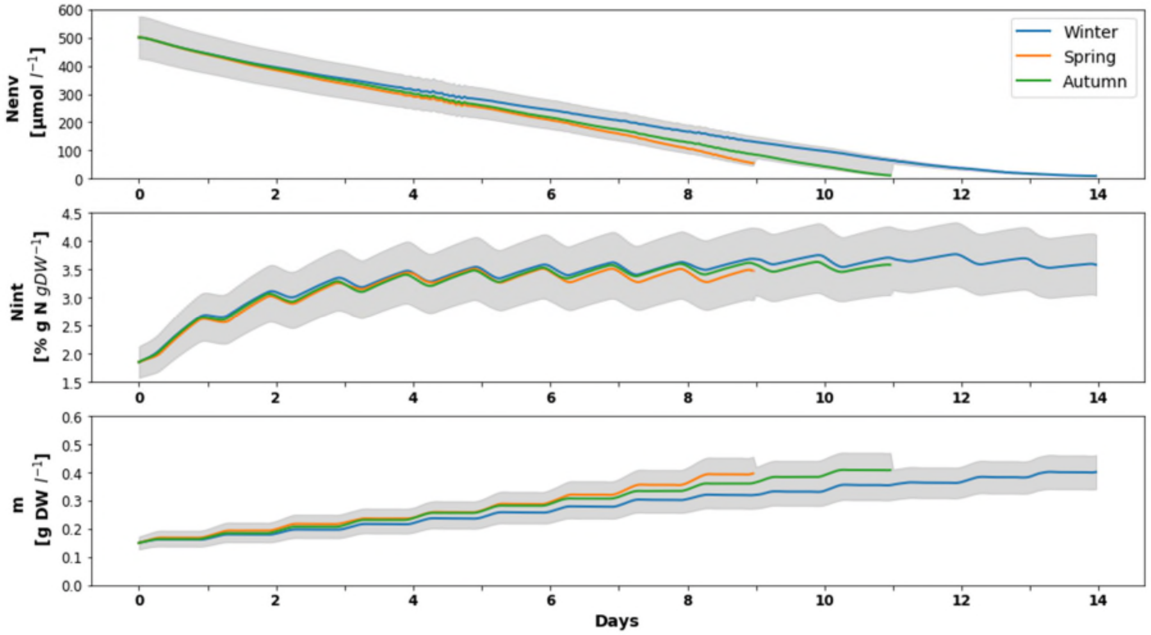

Simulated cultivation periods for the last reactor in a farm of 731 cages: 14 days in winter (blue), 9 days in spring (orange) and 11 days in autumn (green). Shaded region represents a 15% two-side calibration error.

Supplementary Table 1. Metabolic model governing equations

State Variable	ODE	Equation	Annotation
m	$\frac{\partial m}{\partial t} = (\mu - \lambda)\, m$ IC: $m_{(t=0)} = m_0$	(eq S1)	μ is biomass specific growth rate as a function of I, T, S and nutrients [1] (eq S1.1 and S1.3-S1.6) and λ is biomass specific losses rate as a function of T (eq S1.2).
N_{int}	$\frac{\partial N_{int}}{\partial t} = \psi_{N_{ext}} - N_{\text{int}}\mu\text{m}$ IC: $N_{\text{int}\ (t=0)} = N_{\text{int}_0}$	(eq S2)	$\psi_{N_{ext}}$ is the N uptake function (eq S2.1) and $N_{\text{int}} f \text{m}$ represents dilution of internal N by growth.
N_{ext}	$\frac{\partial N_{ext}}{\partial t} = -\frac{\nabla\cdot(\mathbf{v}N_{ext})}{V_{cage}} - \psi_{N_{ext}} m$ IC: $N_{\text{ext}\ (t=0)} = N_{\text{ext}_0}$	(eq S3)	$-\frac{\nabla\cdot(\mathbf{v}N_{ext})}{V_{cage}}$ represents the convection of N_{ext} into or out of the reactor and $\psi_{N_{ext}} m$ represents the N sink in the biomass. Eq S3 derives from the Convection-Diffusion equation [12] as described below (eq S3.1).

IC: initial condition

Supplementary Table 2. Experimental data used for model calibration [14]

Return	Starting date (2017)	Final date (2017)	Initial biomass* [g FW m^{-3}]	Final biomass* [g FW m^{-3}]	Initial N_{int} [% g N g DW^{-1}]	Final N_{int} [% g N g DW^{-1}]	N_{ext} [μmol N l^{-1}]	Light intensity data source
1	20 April	3 May	1,315	4,590	N/A, 2**	1.05	90	Ex-situ
2	3 May	17 May		2,781	1.05	0.65	111	In + Ex-situ
3	17 May	29 May		1,120	0.65	0.98	N/A, 91***	In + Ex-situ
4	15 June	29 May	1,306	2,001	N/A, 3**	1.06	148	In + Ex-situ
5	28 June	12 July		1,069	1.06	1.40	206	In + Ex-situ

*DW:FW conversion ratio of 0.15

**Assumed based on personal communication (A. Chemodanov, April 2020)

*** Used final N_{ext} values instead of N/A initial values

Supplementary Table 3. Simulated environmental conditions. Based on data from the Alexander estuary [15]

Parameter	Unit	Value
Dissolved N	μmol N l^{-1}	500
Dissolved P	μmol P l^{-1}	50
Bottom Salinity	PSU	12-30*
Stream flow	l $hour^{-1}$	7776**
Estuary width	m	22
Estuary depth	m	2.5

*Used a constant salinity value of 30 PSU

**Calculated based on a baseflow controlled by a stable influx of 11.5 10^3 m^3 day^{-1}, normalized to a per hour flow through an area equal to the narrow-side cage cross section (0.89 m^2), in defined estuary width and depth.

Supplementary Table 4. Model parameters: literature values, examined ranges and chosen values

Parameter	Literature values	Unit	Reference	Examined range	Best value	unit	Determining Analysis
μ_{max}	0.416 (0.027)	day^{-1} ($light\ h^{-1}$)	[8]	0.025-0.035	*	Light h^{-1}	
λ_{20}	0.066 (0.0049)	day^{-1} ($light\ h^{-1}$)	[8]	0.014-0.068 (0.001-0.005)	0.022 (0.0016)	day^{-1} (Light h^{-1})	2
θ	1.047	unitless	[1]	0.9-1.2	*		
$N_{\text{int max}}$	4.2	% Kg N Kg DW^{-1}	**	3.2-4.5	*		
$N_{\text{int min}}$	0.7	% Kg N Kg DW^{-1}	[1]	0.5-0.7	*		
$N_{\text{int } crit}$	2	% Kg N Kg DW^{-1}	[16]	1.5-3	*		
V_{max}	60	µmol N g DW^{-1} h^{-1}	[17–19]	50-250	*		
K_S	14	mmol N m^{-3}	[18,20]	10-30	*		
K_I	144.8	µmol photons m^{-2} s^{-1}	[8]	15-150	20	µmol photons m^{-2} s^{-1}	2
K_0	1.5	m^{-1}	[8]	0.1-3	*		
K_a	0.01	m^2 gDW^{-1}	[8]	0.01-0.2	0.15	m^2 gDW^{-1}	1
T_{min}	5	℃	[1]	1-10	*		
T_{opt}	25	℃	[1]	15-20	18	℃	
T_{max}	35	℃	[1]	29-32	31.5	℃	
n	2	-	[1]	1-6	2		2
S_{min}	0	PSU	[1]	0-10	*		
S_{opt}	18	PSU	[1]	15-25	*		
S_{max}	45	PSU	[1]	40-50	*		
Q_P	460	l h^{-1}	[14]	300-600	*		
d	0	% $reactor^{-1}$		0-5	*		

*kept original literature/assumed value unchanged
**$N_{\text{int max}}$ value determined based on personal observations
DW: dry weight

Supplementary Table 5. Calibration results: measured and modeled biomass production in returns 1,2,4 and 5

Return	Initial biomass [g FW m^{-3}]	Measured Final biomass [g FW m^{-3}]	Modeled Final biomass: In-Situ data [g FW m^{-3}]	Modeled Final biomass: Ex-Situ data [g FW m^{-3}]	Relative Error 1*	Relative Error 2**
1	1,315	4,590		3,961		0.137
2		2,781	3,518	3,137	0.121	0.128
4	1,306	2,001	1,810	1,602	0.129	0.200
5		1,069	1,064	1,048	0.017	0.021
RMSRE					0.103	0.138

*Calculated by eq S8

**Calculated by eq S9

RMSRE: root mean square relative error

Supplementary References

1. Lehahn, Y., Ingle, K. N. & Golberg, A. Global potential of offshore and shallow waters macroalgal biorefineries to provide for food, chemicals and energy: feasibility and sustainability. *Algal Res.* **17**, 150–160 (2016).
2. Lemesle, V. & Mailleret, L. A Mechanistic Investigation of the Algae Growth "Droop" Model. *Acta Biotheor.* **56**, 87–102 (2008).
3. Martins, I. & Marques, J. C. A Model for the Growth of Opportunistic Macroalgae (Enteromorpha sp.) in Tidal Estuaries. *Estuar. Coast. Shelf Sci.* **55**, 247–257 (2002).
4. Zollmann, M., Traugott, H., Chemodanov, A., Liberzon, A. & Golberg, A. Deep Water Nutrient Supply for an Offshore Ulva sp. Cultivation Project in the Eastern Mediterranean Sea: Experimental Simulation and Modeling. *Bioenergy Res.* **12**, 1113–1126 (2019).
5. Fort, A. *et al.* Extensive variations in diurnal growth patterns and metabolism among ulva spp. Strains. *Plant Physiol.* **180**, 109–123 (2019).
6. Martins, I. & Marques, J. C. A model for the growth of opportunistic macroalgae (Enteromorpha sp.) in tidal estuaries. *Estuar. Coast. Shelf Sci.* **55**, 247–257 (2002).
7. Choi, T. S., Kang, E. J., Kim, J.-H. & Kim, K. Y. Effect of salinity on growth and nutrient uptake of Ulva pertusa (Chlorophyta) from an eelgrass bed. *Algae* **25**, 17–26 (2010).
8. Oca, J., Cremades, J., Jiménez, P., Pintado, J. & Masaló, I. Culture of the seaweed Ulva ohnoi integrated in a Solea senegalensis recirculating system: influence of light and biomass stocking density on macroalgae productivity. *J. Appl. Phycol.* **31**, 2461–2467 (2019).
9. Ménesguen, A., Cugier, P. & Leblond, I. A new numerical technique for tracking chemical species in a multi-source, coastal ecosystem, applied to nitrogen causing Ulva blooms in the Bay of Brest (France). *Limnol. Oceanogr.* **51**, 591–601 (2006).
10. Hanisak, M. D. Nitrogen Limitation of Codium fragile ssp. tomentosoides as Determined by Tissue Analysis. *Mar. Biol.* **50**, 333–337 (1979).
11. Harrison, P. J., & Hurd, C. L. Nutrient physiology of seaweeds: application of concepts to aquaculture. *Cah. Biol. Mar.* 1–2 (2001).
12. Thomas, S. *Introduction to Climate Modelling*. (Springer Science & Business Media, 2011).
13. Mottus, M., Sulev, M., Baret, F., Reinart, A. & Lopez, R. Photosynthetically Active Radiation : Measurement and Modeling. (2011).
14. Chemodanov, A. *et al.* Feasibility study of *Ulva* sp. (Chlorophyta) intensive cultivation in a coastal area of the Eastern Mediterranean Sea. *Biofuels, Bioprod. Biorefining* **13**, 864–877 (2019).
15. Suari, Y. *et al.* Sandbar Breaches Control of the Biogeochemistry of a Micro-Estuary RIME-restoration of Israeli micro estuaries View project Effect of water circulation on the Yarqon River ecosystem View project. *Front. Mar. Sci.* (2019). doi:10.3389/fmars.2019.00224
16. Pedersen, M. F. & Borum, J. Nutrient control of algal growth in estuarine waters. Nutrient limitation and the importance of nitrogen requirements and nitrogen storage among phytoplankton and species of macroalgae. *Mar. Ecol. Prog. Ser. Ser.* **142**, 261–272 (1996).
17. Jing-Wen, L. & Shuang-Lin, D. Comparative studies on utilizing nitrogen capacity between two macroalgae Gracilaria tenuistipitata var. liui (rhodophyta) and Ulva pertusa (chlorophyta)Ⅱ.Feedback controls of intracellular nitrogen pools on nitrogen uptake. *J. Environ. Sci.* **13**, 323–327 (2001).
18. Pedersen, M. F. TRANSIENT AMMONIUM UPTAKE IN THE MACROALGA ULVA LACTUCA (CHLOROPHYTA): NATURE, REGULATION, AND THE CONSEQUENCES FOR CHOICE OF MEASURING TECHNIQUE1. *J. Phycol.* **30**, 980–986 (1994).
19. Gevaert, F., Barr, N. G. & Rees, T. A. V. Diurnal cycle and kinetics of ammonium assimilation in the green alga Ulva pertusa. *Mar. Biol.* **151**, 1517–1524 (2007).
20. Bay, P. & Campbell, S. J. Uptake of ammonium by four species of macroalgae in Port Phillip Bay, Victoria, Australia. *Mar. Freshw. Res.* **50**, 515–522 (1999).
