# Supplementary material for: Multi-scale modeling of intensive macroalgae cultivation and marine nitrogen sequestration
Source: Commun Biol. 2021 Jul 7;4:848. doi: 10.1038/s42003-021-02371-z (PMC8263761; doi:10.1038/s42003-021-02371-z)
Supplement: Supplementary file 3 — Reporting Summary [file 42003_2021_2371_MOESM3_ESM.pdf]

nature research

Corresponding author(s): Meiron Zollmann

Last updated by author(s): 31/5/21

Reporting Summary

Nature Research wishes to improve the reproducibility of the work that we publish. This form provides structure for consistency and transparency in reporting. For further information on Nature Research policies, see our Editorial Policies and the Editorial Policy Checklist.

Statistics

For all statistical analyses, confirm that the following items are present in the figure legend, table legend, main text, or Methods section.

n/a	Confirmed	
[ ]	[x]	The exact sample size (n) for each experimental group/condition, given as a discrete number and unit of measurement
[ ]	[x]	A statement on whether measurements were taken from distinct samples or whether the same sample was measured repeatedly
[x]	[ ]	The statistical test(s) used AND whether they are one- or two-sided<br>*Only common tests should be described solely by name; describe more complex techniques in the Methods section.*
[x]	[ ]	A description of all covariates tested
[ ]	[x]	A description of any assumptions or corrections, such as tests of normality and adjustment for multiple comparisons
[x]	[ ]	A full description of the statistical parameters including central tendency (e.g. means) or other basic estimates (e.g. regression coefficient) AND variation (e.g. standard deviation) or associated estimates of uncertainty (e.g. confidence intervals)
[x]	[ ]	For null hypothesis testing, the test statistic (e.g. F, t, r) with confidence intervals, effect sizes, degrees of freedom and P value noted<br>*Give P values as exact values whenever suitable.*
[x]	[ ]	For Bayesian analysis, information on the choice of priors and Markov chain Monte Carlo settings
[x]	[ ]	For hierarchical and complex designs, identification of the appropriate level for tests and full reporting of outcomes
[x]	[ ]	Estimates of effect sizes (e.g. Cohen's d, Pearson's r), indicating how they were calculated

Our web collection on statistics for biologists contains articles on many of the points above.

Software and code

Policy information about availability of computer code

Data collection	Code was not used to collect data
Data analysis	Data analysis and computer simulations were performed in Python 3

For manuscripts utilizing custom algorithms or software that are central to the research but not yet described in published literature, software must be made available to editors and reviewers. We strongly encourage code deposition in a community repository (e.g. GitHub). See the Nature Research guidelines for submitting code & software for further information.

Data

Policy information about availability of data

All manuscripts must include a data availability statement. This statement should provide the following information, where applicable:

- Accession codes, unique identifiers, or web links for publicly available datasets
- A list of figures that have associated raw data
- A description of any restrictions on data availability

The entire code and data of this study is available as an open source in https://doi.org/10.5281/zenodo.4062432.

Field-specific reporting

Ecological, evolutionary & environmental sciences study design

All studies must disclose on these points even when the disclosure is negative.

Study description	In this study we develop and calibrate a multi-scale model for Ulva sp. macroalgae growth and nitrogen bio-sequestration and use it for farm-scale simulations under different conditions. Model parameters were calibrated by using data from four field cultivation experiments (n=4) and examining the root mean square relative error of empiric results vs model predictions of 600 parametric combinations. The sensitivity of the model to the different parameters was analyzed using 420 random parametric combinations of all model parameters. Model simulations include seasonal changes in biomass production and nitrogen sequestration and how are these variables affected by water exchange in the reactor scale and dilution in the farm scale.
Research sample	The study used previously reported results from four Ulva sp. near-shore cultivation experiments for model calibration.<br>All data used for model calibration was taken from:<br>Chemodanov, A., Robin, A., Jinjikhashvily, G., Yitzhak, D., Liberzon, A., Israel, A., & Golberg, A. (2019). Feasibility study of Ulva sp. (Chlorophyta) intensive cultivation in a coastal area of the Eastern Mediterranean Sea. Biofuels, Bioproducts and Biorefining, 13(4), 864-877.
Sampling strategy	The study did not include sampling. Model calibration was done using the available data from the simulated cultivation system. Return #3 (out of 5) was not used for calibration as its negative growth could not be explained by the model
Data collection	Data was collected from a few sources:<br>1. Cultivation data, including light intensity and temperature measurement by a HOBO device, all used for model calibration, was recorded by Alexander Chemodanov and published in: Chemodanov, A., Robin, A., Jinjikhashvily, G., Yitzhak, D., Liberzon, A., Israel, A., & Golberg, A. (2019). Feasibility study of Ulva sp.(Chlorophyta) intensive cultivation in a coastal area of the Eastern Mediterranean Sea. Biofuels, Bioproducts and Biorefining, 13(4), 864-877.<br>2. Data from the Alexander estuary, used for model simulations, were collected by Yair Suari and his team and published in: Suari, Y. et al. Sandbar Breaches Control of the Biogeochemistry of a Micro-Estuary RIME-restoration of Israeli micro estuaries View project Effect of water circulation on the Yarqon River ecosystem View project. Front. Mar. Sci. (2019). doi:10.3389/fmars.2019.00224<br>3. Light intensity and air temperature data used for simulations was extracted from the Israeli Meteorological Service: https://ims.gov.il/he/ObservationDataAPI
Timing and spatial scale	The model developed in this study was calibrated using data collected in five consecutive experiments between April 20 and July 12, 2017. This is the period in which the near-shore experiments in the simulated system took place. The scale of these experiments was 1m*1m*2m. Model simulations used data from 2014. Water temperature was measured once a month while air temperature and light intensity was measured every 1-3 hours.
Data exclusions	Model calibration was performed with growth data (except from return 3, in which growth was negative), without relating to external and internal nitrogen measurements. The data of internal and external nitrogen was not complete (i.e not measure continuously). For this reason, and due to our understanding that the cultivation environment was P limited, which affects nitrogen utilization, we chose to calibrate the model only with growth measurement, which were complete and 100% reliable.<br>This is mentioned in the manuscript as a limitation of the study and as a future study needed for improved model calibration
Reproducibility	The focus of the paper is the developed model and the simulations it can produce. The experimental findings were published in a previous paper and are beyond the scope of this work: Chemodanov, A., Robin, A., Jinjikhashvily, G., Yitzhak, D., Liberzon, A., Israel, A., & Golberg, A. (2019). Feasibility study of Ulva sp.(Chlorophyta) intensive cultivation in a coastal area of the Eastern Mediterranean Sea. Biofuels, Bioproducts and Biorefining, 13(4), 864-877.<br>As mentioned above, further calibrations and validations of the model will support and improve the model. We are working on additional robust experimental validation in current works.
Randomization	Randomization of parameters was performed in the calibration stage and in the sensitivity analysis, using the Saltelli method.
Blinding	Blinding was not relevant to this work. We calibrated the developed model with the existing data and explained the limitation of this calibration

Did the study involve field work? ☐ Yes ☒ No

Reporting for specific materials, systems and methods

We require information from authors about some types of materials, experimental systems and methods used in many studies. Here, indicate whether each material, system or method listed is relevant to your study. If you are not sure if a list item applies to your research, read the appropriate section before selecting a response.

Materials & experimental systems

n/a	Involved in the study
☒	☐ Antibodies
☒	☐ Eukaryotic cell lines
☒	☐ Palaeontology and archaeology
☒	☐ Animals and other organisms
☒	☐ Human research participants
☒	☐ Clinical data
☒	☐ Dual use research of concern

Methods

n/a	Involved in the study
☒	☐ ChIP-seq
☒	☐ Flow cytometry
☒	☐ MRI-based neuroimaging
